# Calpain3 Is Expressed in a Proteolitically Active Form in Papillomavirus-Associated Urothelial Tumors of the Urinary Bladder in Cattle

**DOI:** 10.1371/journal.pone.0010299

**Published:** 2010-04-22

**Authors:** Sante Roperto, Roberta De Tullio, Cinzia Raso, Roberto Stifanese, Valeria Russo, Marco Gaspari, Giuseppe Borzacchiello, Monica Averna, Orlando Paciello, Gianni Cuda, Franco Roperto

**Affiliations:** 1 Department of Pathology and Animal Health, Division of Infectious Diseases, Naples University Federico II, Naples, Italy; 2 Department of Experimental Medicine (DIMES), Biochemistry Section and Centre of Excellence for Biomedical Research (CEBR), University of Genova, Genova, Italy; 3 Department of Experimental Medicine and Clinics, University of Catanzaro ‘Magna Graecia’, Catanzaro, Italy; 4 Department of Pathology and Animal Health, Division of General Pathology, Naples University Federico II, Naples, Italy; University of Pretoria, South Africa

## Abstract

**Background:**

Calpain 3 (Capn3), also named p94, is a skeletal muscle tissue-specific protein known to be responsible for limb-girdle muscular dystrophy type 2A (LGMD2A). Recent experimental studies have hypothesized a pro-apoptotic role of Capn3 in some melanoma cell lines. So far the link between calpain3 and tumors comes from *in vitro* studies. The objective of this study was to describe Capn3 activation in naturally occurring urothelial tumors of the urinary bladder in cattle.

**Methods and Findings:**

Here we describe, for the first time in veterinary and comparative oncology, the activation of Capn3 in twelve urothelial tumor cells of the urinary bladder of cattle. Capn3 protein was initially identified with nanoscale liquid chromatography coupled with tandem mass spectrometry (nano LC-MS/MS) in a co-immunoprecipitation experiment on E2F3, known to be a transcription factor playing a crucial role in bladder carcinogenesis in humans. Capn3 expression was then confirmed by reverse transcription polymerase chain reaction (RT-PCR). Finally, the Ca^2+^-dependent proteolytic activity of Capn3 was assayed following ion exchange chromatography. Morphologically, Capn3 expression was documented by immunohistochemical methods. In fact numerous tumor cells showed an intracytoplasmic immunoreactivity, which was more rarely evident also at nuclear level. In urothelial tumors, bovine papillomavirus type 2 (BPV-2) DNA was amplified by PCR and the expression of E5 protein, the major oncogenic protein of BVP-2, was detected by western blotting, immunohistochemistry, and immunofluorescence. E2F3 overexpression and pRb protein downregulation were shown by western blotting.

**Conclusion:**

The role of capn3 protein in urothelial cancer of the urinary bladder remains to be elucidated: further studies would be required to determine the precise function of this protease in tumor development and progression. However, we suggest that activated Capn3 may be involved in molecular pathways leading to the overexpression of E2F3, which in turn could be responsible for urothelial tumor cell proliferation also in cattle, though other mechanisms are likely to exist. If further studies corroborate the important role of Capn3 in urothelial tumors of the urinary bladder, cattle with urinary tumors may prove useful as animal model for bladder carcinogenesis.

## Introduction

The calpain system is composed of a family of Ca^2+^-dependent cysteine proteases, their activity being regulated by cytosolic calcium [1], [2]. Calpains originally comprised two proteases, calpain 1 (Capn1) or µ-calpain (micromolar) and calpain 2 (Capn2) or m-calpain (millimolar). Also referred to as the “conventional” or “ubiquitous” calpains, they are specifically inhibited by calpastatin [1]. Calpains appear to be involved in cell motility, signal transduction, cell cycle progression, gene expression regulation and apoptosis [1], [3]. Calpains are known to play an important role in macroauthophagy, being regulators of autophagosome formation [4], [5], and in some viral oncogenetic mechanisms [6]. Furthermore, calpains represent a promising target for cancer therapy, since they appear to play a key role in metastatic cell migration and angiogenesis [7].

Calpain 3 (Capn3), also known as calpain p94, is a member of the so-called tissue-specific subfamily, being predominantly expressed in skeletal muscle. Its structure is similar to that of the other members of this family but it presents three specific sequences not found in any other calpain: a novel sequence (NS) at the N-terminus, insertion sequence 1 (IS1) within the catalytic domain, and insertion sequence 2 (IS2) upstream of the Ca^2+^-binding domain [8]. It has been suggested that Capn3 may play a role in sarcomere remodelling and in mitochondrial protein turnover [9]. Point mutations in the Capn3 gene are responsible for limb girdle muscular dystrophy type 2A (LGMD2A), an autosomal recessive disease characterized by progressive atrophy and weakness of the proximal limb muscle [10].

Furthermore, several variants of Capn3 have been reported in many tissues [11] including the eye [12], [13], peripheral blood mononuclear cells [14], and astrocytes [15], thus suggesting that Capn3 is important for tissues other than skeletal muscle [2]. Recently, novel Capn3 isoforms in white blood cells have been sequenced and are being investigated to develop a new approach for performing the molecular diagnosis of LGMD2A at mRNA level [16].

To our knowledge, no investigation has so far been carried out on Capn3 expression in veterinary and comparative spontaneous carcinogenesis.

The aim of the present paper is to report, for the first time in medical literature, the activation of the Capn3 protease in urothelial tumors of the urinary bladder in cattle. Such tumors are very commonly found in adult cattle grazing on lands rich in bracken fern. Furthermore, a strong relationship between bracken fern and bovine papillomavirus type 2 (BPV-2) has been established [17], so that BPV-2 infection appears to be a pivotal event in the bladder carcinogenesis of cattle [17], [18], [19].

## Methods

### Ethics Statement

In our cases we didn't perform any experimentation as we collected tissue samples directly in public slaughterhouses. All the animals we studied were slaughtered after a mandatory clinical *ante-mortem* examination, as required by European Union legislation.

### Bladder samples

Samples of bladder neoplastic urothelium were collected at public slaughterhouses from twelve 4- to 24-year-old cows that had suffered from chronic enzootic hematuria for several years. Samples of bladder mucosa without any apparent gross lesions due to tumor proliferations were also obtained from the same animals. All animals had been raised in hilly/mountain cattle households in the South of Italy and were known to have grazed on pastures rich in bracken fern. Normal bladder mucosa was obtained from five 4- to 14-year-old healthy cows which had grazed on pastures in which no bracken was present. Bladder samples were routinely divided into four parts. One part was fixed in 10% buffered formalin. Two parts were immediately frozen in liquid nitrogen, stored at −80°C and utilized for molecular procedures. The remaining part was frozen in isopentane pre-cooled in liquid nitrogen and stored at −80°C until further processed for immunohistochemistry.

### Histopathology

The tissues fixed in 10% buffered formalin were routinely processed for paraffin embedding. Histologic diagnosis was assessed on 5-µm-thick haematoxylin-eosin (HE)–stained sections using morphological criteria suggested in a recent report on the new histological classification of urothelial tumors of the urinary bladder of cattle [20].

### E2F3 immunoprecipitation for proteomic analysis

E2F3 overexpression is known to play a crucial role in bladder carcinogenesis [21]. To investigate its potential molecular partners, a co-immunoprecipitation experiment with E2F3 was performed. Tissues were lysed in ice-cold buffer containing 50 mM Tris-HCl (pH 7.5), 1% (v/v) Triton X-100, 150 mM NaCl, 2 mM PMSF, 1.7 mg/ml Aprotinin, 50 mM NaF, and 1 mM sodium orthovanadate. Lysates were clarified by centrifugation at 11,000 g for 30 minutes. Supernatants were collected, and protein concentration was determined by a modified Bradford assay (Bio-Rad). One mg per sample of proteins was immunoprecipitated using, in a first step, IgG mouse (Sigma) with 30 µl of G-sepharose (Ge Healthcare) for preclearing, and, in a second step, 2 µg of anti-E2F3 antibody (Upstate) with the same amount of G-sepharose. Immunoprecipitates were washed four times in a complete lysis buffer and finally heated in Laemmli buffer composed of glycerol 40% (Sigma), β-mercaptoethanol 0,35 M (Sigma), SDS 5% (Sigma), and Blue Bromophenol (Roche). Immunoprecipitates were separated on 4–12% polyacrylamide gels and then submitted to in-gel digestion.

### In-gel digestion of IP protein bands

Gel bands for mass spectrometric analysis were basically processed according to Shevchenko *et al.* [22]. Sliced gel pieces were washed with 100 mM NH_4_HCO_3_ and acetonitrile (1∶1, v/v) (buffer A). HPLC-grade acetonitrile was obtained from Sigma-Aldrich (St. Louis, MO). Proteins were in-gel reduced by 10 mM DTT, and subsequently alkylated with 20 mM iodoacetamide. After a washing step with buffer A, the gel pieces were dried in a vacuum centrifuge, and rehydrated at 4°C in a digestion buffer (50 mM NH_4_HCO_3_, 5 mM CaCl_2_) containing 25 ng/µl trypsin. After overnight incubation, peptides were extracted from the gel using three separate washings with a mixture of acetonitrile/water/formic acid 70/25/5 (v/v/v). Extracts were combined and dried down in a vacuum centrifuge.

The lyophilized digests were reconstituted in 30 µl of loading pump solvent (see Nanoscale LC-MS/MS Section). An aliquot of the solution (10 µl) was then injected for nanoscale LC-MS/MS analysis.

### Nanoscale Liquid Chromatography coupled with tandem Mass Spectometry (nano LC-MS/MS)

Chromatography was performed on an Ultimate nano LC system from Dionex (Sunnyvale, CA). The analytical nano LC column used was an in-house packed 75 µm i.d., 40 cm long Integra Frit™, column from New Objective (Cambridge, MA), filled with 4 µm C_12_ silica particles Jupiter Proteo from Phenomenex (Torrence, CA); a mixture of the peptide (10 µL) was loaded onto an in-house packed 150 µm i.d., 3 cm long Integra Frit™ (New Objective) trapping column (packing bed length 1 cm) at 12 µL/min of loading pump solvent, consisting of H_2_O/acetonitrile/trifluoroacetic acid (TFA) 97.95:2:0.05 (v/v/v). After a 2-minute washing, the trapping column was switched on-line to the analytical column, and gradient separation started at 200 nL/min.

A binary gradient was used for peptide elution. Mobile phase A was H_2_O/acetonitrile/formic acid/TFA 97.9:2:0.09:0.01 (v/v/v/v); mobile phase B was H_2_O/acetonitrile/formic acid/TFA 29.9:70:0.09:0.01 (v/v/v/v). Gradient was from 5 to 45% B in 60 minutes at 200 nL/min flow rate. After 10 minutes at 95% B, the column was re-equilibrated at 5% B for 30 minutes before injection. MS detection was performed on a QSTAR XL hybrid LC-MS/MS from Applied Biosystems (Foster City, CA) operating in positive ion mode, with nESI potential at 1800 V, curtain gas at 15 units, CAD gas at 3 units. Nanoelectrospray ionization was achieved via distal coated Pico Tips™ 20 µm ID, 10 µm tip ID (New Objective). Information-dependent acquisition (IDA) was performed by selecting the two highest peaks for MS/MS analysis after a full TOF-MS scan from 400 to 1600 m/z lasting 4 seconds. Both MS/MS analyses were performed in enhanced mode (3 second/scan). Threshold value for peak selection for MS/MS was 20 counts.

### RNA isolation, cDNA synthesis, and PCR

Total RNA was isolated both from normal and neoplastic mucosa. Furthermore, RNA was also isolated from the same neoplastic bladders in an apparently unaffected mucosa region (since it did not show any gross neoplastic lesions), according to manufacturer's instructions (Paris kit, Ambion). cDNAs were synthesized from bovine bladders or human PBMC starting from total RNA (4 µg), using Thermoscript RT-PCR system (Invitrogen) [14]. Calpain 3 transcript was amplified from cDNAs using PCR_x_ Taq Polymerase Enhancer System (Invitrogen). A pair of primers flanking a region of the protease sequence known to contain calpain 3 (Bos Taurus calpain 3 p94, CAPN3 GenBank Accession No NM_174260.2) specific insertion sequence 1 (IS1) were used: forward primer (Sn 831) 5′-CTGCTGGAGAAGGCTTATGC-3′, reverse primer (Asn 1845) 5′-CCCGCATGTTGATGTAGGTT-3′. Another pair of primers flanking a region of the protease sequence known to contain calpain 3 specific insertion sequence 2 (IS2) was also used: forward primer (Sn 1884) 5′-GTCATCGTGCCCTCCACC-3′, reverse primer (Asn 2669) 5′-TCAGGCATACATGGTGAGCTGCAG-3′. PCR was performed using the following parameters: a denaturation step for 2 min at 98°C; then 95°C for 40 s, 55°C for 40 s and 72°C for 2 min, for 40 cycles. To determine the relative levels of calpain 3 transcript in different animals, equal amounts of each cDNA sample were amplified in the presence of primers (forward primer 5′-ACGACCCCTTCATTGACC-3′, reverse primer 5′-TGCTTCACCACCTTCTTG) specific for glyceraldehyde-3-phosphate dehydrogenase (GAPDH). PCR conditions were the following: a denaturation step for 2 min at 98°C; then 95°C for 30 s, 55°C for 30 s and 72°C for 1 min, for 22 cycles. PCR products were separated by electrophoresis on 1.2% agarose gel (Biorad).

### Detection of calpain 3 activity in bovine bladders

Bovine bladder samples collected as above described were stored at −80°C until further processed. Bladders from healthy or pathological animals were minced, homogenized and lysed by sonication (six bursts, 30 sec each) in five volumes of ice-cold 50 mM Na Acetate buffer pH 6.7, containing 1 mM EDTA, 0.5 mM 2-mercaptoethanol. The particulate material was discarded by centrifugation (100,000 g for 15 min, 4°C) and the soluble fraction (35 mg protein) was loaded onto a ion-exchange DE52 column (4 ml), previously equilibrated in 50 mM Na Acetate buffer pH 6.7 containing 0.1 mM EDTA, 0.5 mM 2-mercaptoethanol. The absorbed proteins were eluted with a linear gradient (70 ml) 0–0.4 M NaCl in 1 ml fractions. Calpain activity was assayed on aliquots (150 µl) of each eluted fraction as previously described [23].

### Immunohistochemical examination

Six µm-sectioned frozen samples were fixed in acetone at 4°C for 5 min, then blocked for endogenous peroxidase in 0.3% H_2_O_2_ in methanol for 20 min. Tissue sections were then incubated overnight at room temperature with an anti-rabbit calpain p94 mouse monoclonal antibody (Chemicon International, Billerica, MA, USA), diluted 1∶100 and 1∶200. Slides were washed three times with PBS, then labelled with streptavidin biotin (LSAB kit; DakoCytomation, Denmark) for 30 min, followed by incubation with streptavidin conjugated to horseradish peroxidase (LSAB Kit; DakoCytomation, Denmark). Color development was obtained following 5–20 min of diaminobenzidine (DakoCytomation, Denmark) treatment. Sections were finally counterstained with Mayer's hematoxylin.

### Viral DNA analysis

A small fragment of frozen tissue of the urothelial tumors was digested by Proteinase-K in a lysis buffer (50 mM KCl, 10 mM Tris HCl, pH 8.3, 2.5 mM MgCl2, 100 µg/ml gelatin, 0.45% NP-40, and 0.45% Tween-20), in order to recover the genomic DNA. Ten microliters of each sample were amplified by PCR utilizing one unit of Taq polymerase (Platinum*Taq* Invitrogen, Milan, Italy) in 50 µl of the buffer provided by the manufacturer with 3 mM MgCl_2_, and 2.5 mM of each dNTP. The reaction was carried out in an iCycler (Bio-Rad Laboratories, Milan, Italy) using the 5′-TACTGTTTCTGCTGCTATTT-3′ forward primer and the 5′-ACAAATCAAATCCACATAATAGTA-3′ reverse primer that amplify a small fragment of BPV-2 (125 bp). PCR conditions were as follows: denaturation for 2 min at 95°C, followed by 35 cycles of denaturation at 95°C for 30 s, annealing at 50°C for 30 s, and extension at 72°C for 1 min [19]. The final PCR products were electrophoresed in 2% agarose gel and visualized by ethidium bromide stain. Each experiment included a blank sample consisting of reaction mixture without DNA and a positive sample consisting of a recombinant plasmid carrying the genomic sequence of BPV-2 (kindly provided by Dr. M. S. Campo, Glasgow University, Scotland). A band corresponding to the size of the amplified sequences of BPV-2 was detected in the examined cancer samples. To confirm the PCR data, the amplified products were purified through silicagel membranes by the QIAquick PCR quantification kit according to the manufacturer's instructions (QIAgen, Milan, Italy) and then subjected to direct sequencing in an automated apparatus (Biogen, Rome, Italy).

### Immunoprecipitation and immunofluorescence for BPV-2 E5 protein

Tissues were lysed in ice-cold buffer containing 50 mM Tris-HCl (pH 7.5), 1% (v/v) Triton X-100, 150 mM NaCl, 2 mM PMSF, 1.7 mg/ml Aprotinin 50 mM NaF, and 1 mM sodium orthovanadate. Lysates were clarified by centrifugation at 10,000 g for 30 minutes. Supernatants were collected, and protein concentration was determined by a modified Bradford assay (Bio-Rad). One mg per sample of proteins was immunoprecipitated using 2 µg of anti-E5 antibody (kindly provided by Dr. Campo, Glasgow University, Scotland) and 30 µl of G-sepharose (Ge Healthcare). Immunoprecipitates were washed four times in complete lysis buffer and finally heated in LDS loading buffer 4X (Invitrogen) at 70°C for 10 minutes according to the manufacturer's protocol. Immunoprecipitates were separated on 4–12% polyacrylamide gels and transferred to nitrocellulose filter membranes (Biorad). Membranes were blocked in 5% nonfat dry milk, incubated with primary antibodies, detected by the appropriate secondary antibodies, and revealed with an enhanced chemiluminescence system (Amersham Biosciences).

For immunofluorescence, paraffin sections were deparaffinized, rehydrated and heated in a microwave oven (twice, for 5 min each at 750 W) to allow antigen to be unmasked. Slides were then incubated with a 1∶50 dilution of rabbit anti-E5 antiserum (kindly provided by Dr Schlegel, Georgetown University, USA) and, thereafter, with a FITC-conjugated secondary antibody (Chemicon). Immunofluorescence was analyzed with a Zeiss LSM 510 laser scanning confocal microscope (Carl Zeiss GmbH, Jena, Germany).

### Western blot analysis

In order to validate protein identification based on a single protein as detected by proteomic approach and also investigate the expression levels of E2F3 and retinoblastoma tumor suppressor protein (pRb), the latter known to regulate E2F activities, western blot analysis was performed. Briefly, tissues were lysed in a buffer containing 50 mM Tris-HCl (pH 7.5), 1% (v/v) Triton X-100, 150 mM NaCl, 2 mM PMSF, 1.7 mg/ml Aprotinin, 50 mM NaF, and 1 mM sodium orthovanadate, using an Ultra Turrax (Ika-Werke). LDS loading buffer 4X (Invitrogen) was added to the protein samples (40 µg) and they were then heated at 70°C for 10 min as indicated in manufacturer's instructions. Electrophoresis of the proteins was carried out in a MOPS 4–20% gradient gel (Invitrogen). Proteins were blotted on PVDF membranes and subsequently incubated with the appropriate antibodies. Protein bands were detected using ECL (Amersham). The following antibodies (Abs) were used: anti-E2F3 mouse monoclonal Ab (Upstate), anti-pRB goat polyclonal Ab (Santa Cruz), anti-serpin3 mouse monoclonal Ab (Sigma), and anti-actin and anti-tubulin mouse monoclonal Abs (Sigma) as controls. Appropriate secondary antibodies were also utilized (Amersham Biosciences).

## Results

### Microscopical morphology of the tumors

The histological patterns of examined tumors were diagnosed as low grade papillary carcinoma (three cases), high grade papillary carcinoma (one case), low grade invasive carcinoma (two cases), high grade invasive carcinoma (two cases), primary carcinoma *in situ* (CIS) (two cases), papillary urothelial neoplasm of low malignant potential (PUNLMP) (one case), and papilloma (one case).

### Proteomic analysis

Nano LC-MS/MS analysis and database search of tryptic peptides generated by in-gel digestion of protein bands immunoprecipitated with E2F3 allowed the identification of the proteins reported in Table 1.

**Table 1:** Proteins identified by nanoLC-MS/MS analysis of IP bands.

Accession	Protein Description	N. Peptides	Peptide sequences*	Theor. Mw (kDa)
AAD05333	Calpain p94	1	MVRNMDNSR(17)	37
Q3SZQ8_BOVINE	SerpinA3-7 (formerly endopin 2B)	1	LAVSHVIHK(30)	47
HSHU4	Histone H4	3	VFLENVIR(34) AVTYTEHAK(33) ISGLIYEETR(40)	11

(*) The Mascot score for each individual peptide is reported in parenthesis. Significance threshold (p<0.05) corresponded to a Mascot score of 15. Protein identifications based on a single peptide above the significance threshold were validated by careful visual inspection of MS/MS data and western blot analysis.

Data were searched on the Mascot search engine (www.matrixscience.com) against the MSDB database (updated in April 2008) using the following parameters: MS tolerance 10 ppm; MS/MS tolerance 0.3 Da; fixed modifications carbamidomethyl cysteine; enzyme trypsin; max. missed cleavages 1; taxonomy *other mammalia*.

Protein hits based on two successful peptide identifications were considered valid. Protein hits based on a single peptide identification with Mascot score higher than the significance level (>15) were retained after manual validation.

### Detection of calpain 3 transcript in cDNAs from bovine bladders

**Figure 1:**
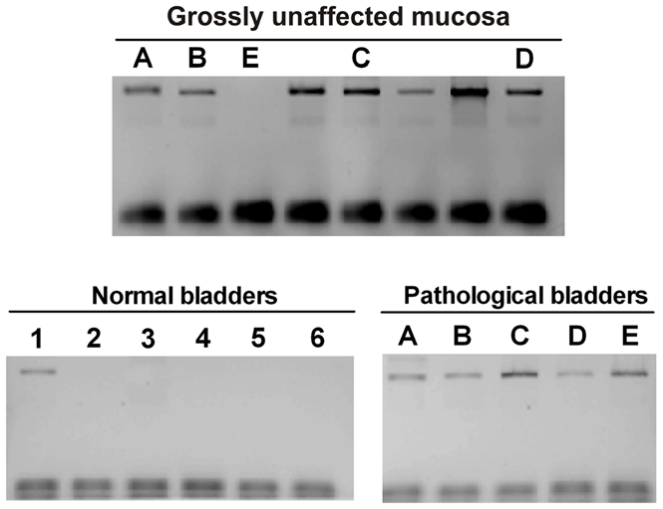
Expression of activated Capn3 in urothelial tumors. Detection of Capn3 transcript in bovine bladders. PCR analysis was performed on the cDNAs synthesized from bladders isolated from different animals as reported in Methods. The primer pair utilized to detect Capn3 was Sn831/Asn1845 and the conditions are reported in Methods. PCR products were separated on 1.2% agarose gel. Each lane corresponds to a different animal. The lanes having the same letter refer to the same animal. cDNA amounts are normalized with respect to equal GAPDH levels.

**Figure 2:**
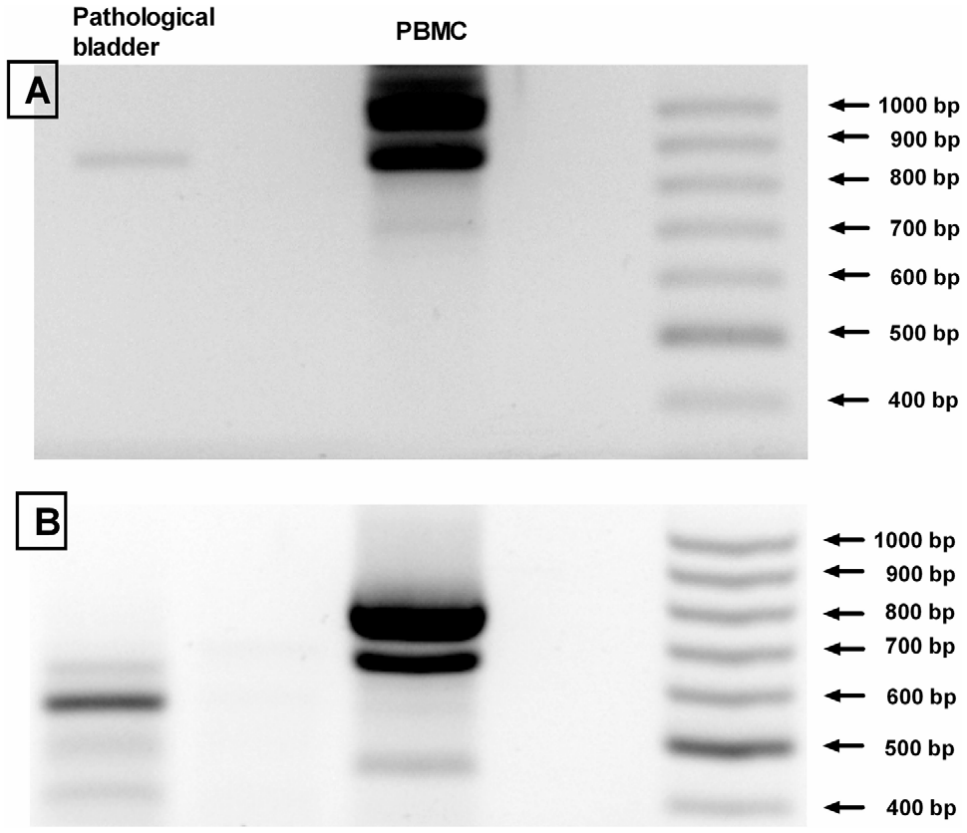
Expression of activated Capn3 in urothelial tumors. Detection of IS1 and IS2 inserts in Capn3 transcript. PCR analysis was performed on the cDNAs synthesized from bladders isolated from pathological animals or human PBMC as reported in Methods. The primer pair utilized to detect Capn3 IS1 insert was Sn831/Asn1845 (A) and that utilized to detect IS2 insert was Sn1884/Asn2669 (B). PCR conditions are reported in Methods. PCR products were separated by electrophoresis on 1.2% agarose gel. Marker sizes: GeneRuler 100 bp DNA ladder (Fermentas). cDNA amounts are normalized with respect to equal GAPDH levels. The expected sizes for the PCR fragments in (A) are 1018 bp with IS1, and 829 bp without IS1. The expected sizes for the PCR fragments in (B) are 805 bp with IS2, and 577 bp without IS2.

The presence of calpain 3 transcript was determined in bovine neoplastic urothelium, on bladder cDNAs obtained from different animals. As shown in Figure 1, all neoplastic bladder samples contained calpain 3 transcripts although at different levels. Theses transcripts were not present in bladders from healthy animals; only in one sample trace amounts of calpain 3 mRNA were detected. We have also observed that calpain 3 transcripts were detectable in apparently unaffected bladder portions, belonging to the same affected animals. Although the amounts of cDNA used for PCR were normalized with respect to GAPDH, quantification of calpain 3 transcripts cannot be precisely determined due to the high number of PCR cycles performed. In fact, the PCR fragment detected following 40 PCR cycles is much less intense in pathological bovine bladders than in human PBMC cDNA, known to express a Capn3-like protease [14] (Figure 2). The data reported in Figure 2 indicate also that pathological bovine bladders express a calpain 3 transcript lacking both IS1 (Figure 2A) and IS2 (Figure 2B) inserts typical of the CAPN3 gene. In conclusion, the calpain 3 expressed in pathological bladders shows peculiar properties due to its low level of transcription and to the absence of IS1 and IS2 structures.

The absence of IS1 internal structure prevents the auto-inactivation step catalyzed by the protease following its exposure to Ca^2+^ [24], whereas the lack of IS2 containing a putative nuclear localization signal, may alter the cellular protease trafficking [25].

### Detection of calpain 3 activity in bovine bladders

**Figure 3:**
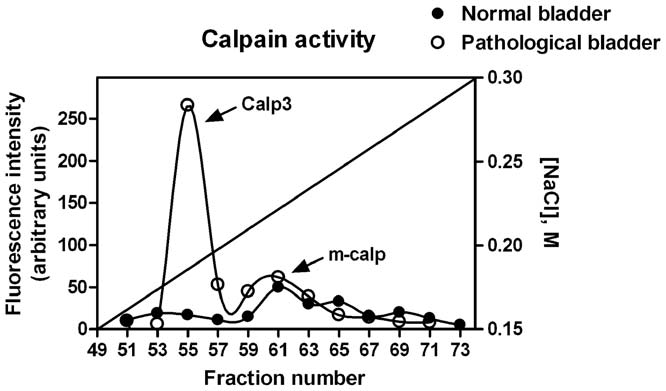
Expression of activated Capn3 in urothelial tumors. Ion-exchange chromatography on bovine bladders. Crude extracts were prepared from normal (full circles) or pathological (empty circles) bovine bladders as reported in methods. Aliquots (35 mg) were submitted to ion-exchange chromatography and calpain activity was assayed on aliquots (100 µl) of the eluted fractions as described in Methods.

To determine the presence of active or activable calpain 3 in the pathological bladder we submitted crude extracts of bovine bladders isolated from both normal and pathological animals to ion-exchange chromatography in order to separate calpain 3 from the ubiquitous µ- and m-calpains, and from calpastatin. As shown in Figure 3, a peak of Ca^2+^-dependent activity eluted from the chromatographic column of the pathological bladder at a ionic strength different from that of µ-, m-calpain and calpastatin. The elution of this calpain 3 protease in this ion-exchange chromatography is slightly different from that previously observed with PBMC-calpain 3 [14], probably because of the difference in the protein structure and in the pH employed in the analysis. In crude extracts from healthy bladders no calpain 3 activity was detectable both in normal and pathological bladders the level of m-calpain (Figure 3) and calpastatin (data not shown) were comparable. Thus, the presence of calpain 3 mRNA and protein is a characteristic feature of these urinary bladder tumors.

**Figure 4:**
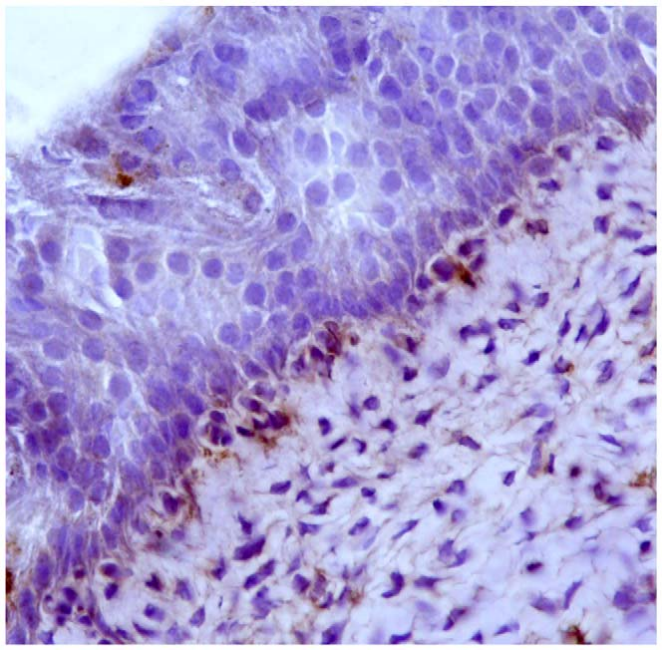
Expression of activated Capn3 in urothelial tumors. Immunohistochemical detection of Capn3. Capn3 is not expressed in normal urothelium. Streptavidin-biotin-peroxidase, Mayer's hematoxylin counterstain.

**Figure 5:**
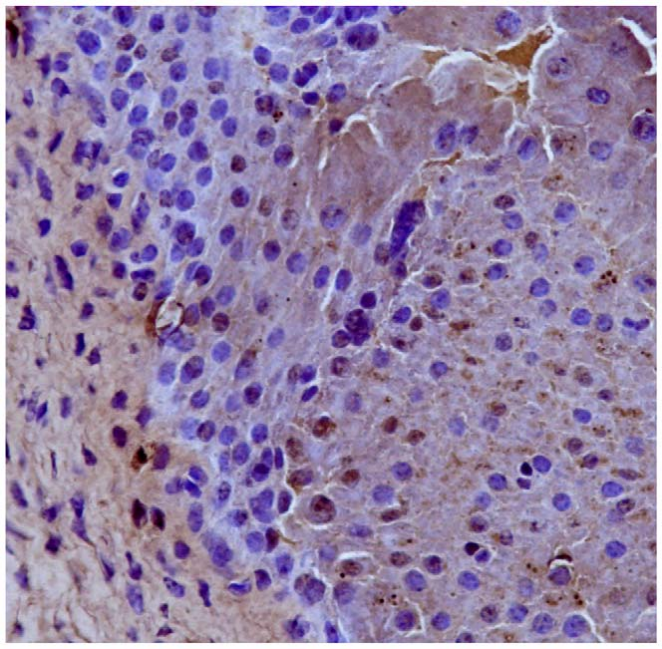
Expression of activated Capn3 in urothelial tumors. A weak immunoreactivity can be seen in urothelial cells of the grossly unaffected mucosa. Streptavidin-biotin-peroxidase, Mayer's hematoxylin counterstain.

**Figure 6:**
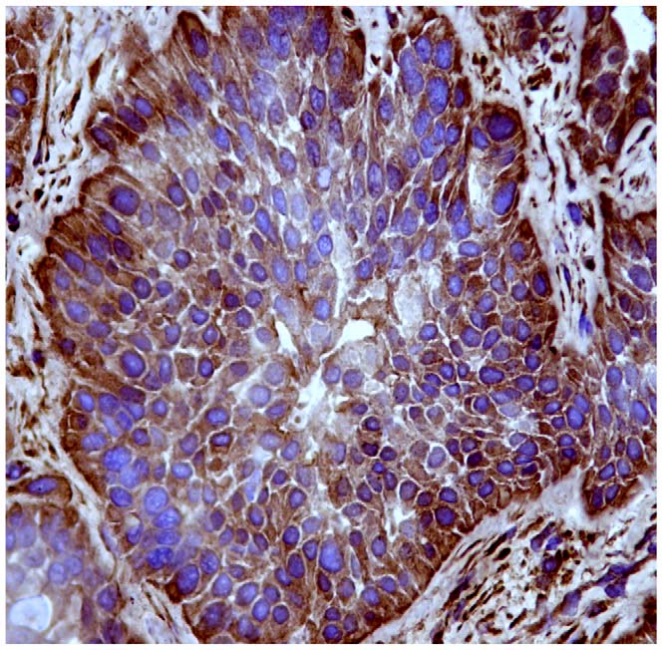
Expression of activated Capn3 in urothelial tumors. Capn3 expression is mostly evident in the cytoplasm of basal neoplastic urothelial cells. Streptavidin-biotin-peroxidase, Mayer's hematoxylin counterstain.

**Figure 7:**
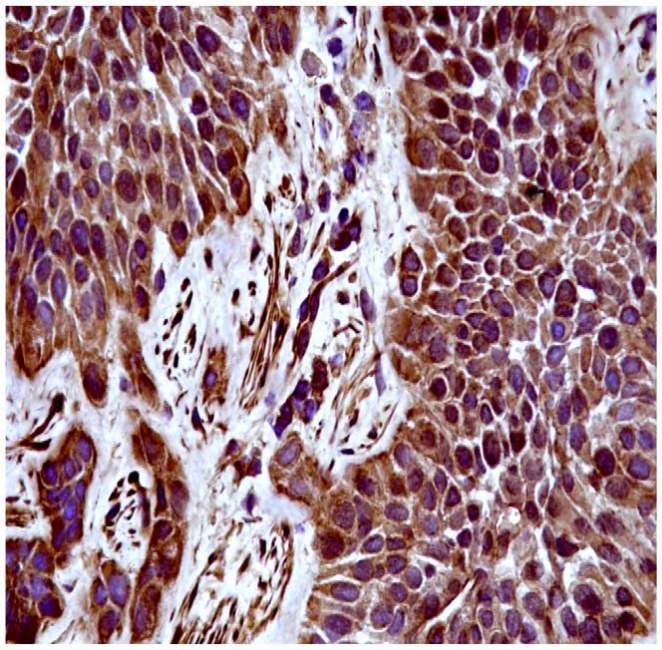
Expression of activated Capn3 in urothelial tumors. Capn3 expression is also detected in some nuclei. Streptavidin-biotin-peroxidase, Mayer's hematoxylin counterstain.

Immunohistochemically, Capn3 was not manifest in normal urothelial cells from healthy cattle (Figure 4). Capn3 was weakly detected in urothelial cells from apparently unaffected mucosa of the neoplastic bladders (Figure 5). In urothelial cancer cells, Capn3 was observed mostly in the cytoplasm. Nuclear positivity was also manifest. Basal cells were the predominant ones showing a strong immunoreactivity for Capn3 (Figures 6 and 7).

### Virus analysis

**Figure 8:**
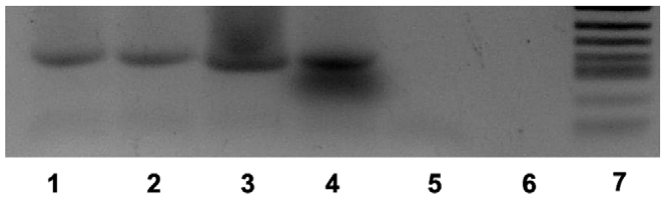
Expression of activated Capn3 in urothelial tumors. PCR amplification of urinary bladder samples. Lanes:1–3, tumor samples; 4, positive control of BPV-2 plasmid; 5–6, negative control with no DNA added; M, molecular mass marker #8 (Roche, Milan, Italy).

BPV-2 DNA was amplified in the tumor samples examined. PCR analysis, validated by direct sequencing of the amplified product, demonstrated the presence of true BPV-2 sequences (Figure 8).

### Immunoprecipitation and immunofluorescence for BPV-2 E5 protein

**Figure 9:**
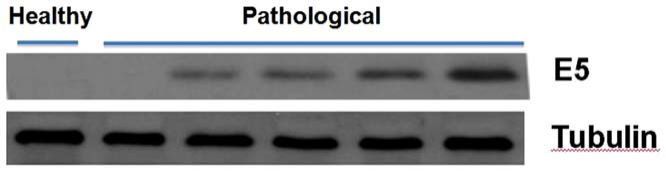
Expression of activated Capn3 in urothelial tumors. E5 oncoprotein expression detected by immunoprecipitation. Lane 1, negative normal bladder tissue from healthy cattle; lanes 2–6 neoplastic samples showing an evident E5 expression except in lane 2.

**Figure 10:**
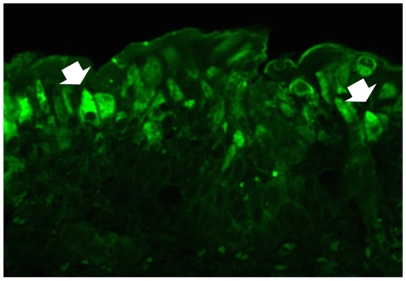
Expression of activated Capn3 in urothelial tumors. E5 oncoprotein documented by scanning laser confocal microscope. Immunofluorescence is evident in the cytoplasm of several neoplastic cells (arrows).

Western blot analysis showed a marked presence of E5 protein in tumor tissue cells (Figure 9). The protein was also present, if only less markedly, in apparently unaffected tissue cells from neoplastic bladders. A cytoplasmic immunofluorescence typical of E5 protein expression was clearly shown in tumor urothelial cells (Figure 10).

### Western blot analysis

**Figure 11:**
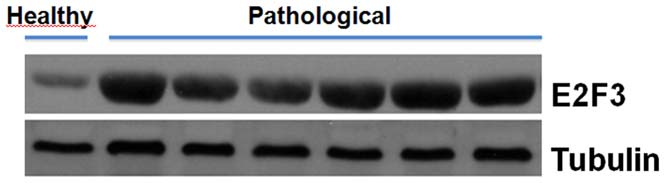
Expression of activated Capn3 in urothelial tumors. Western blot analysis to characterize the expression of E2F3. Lane 1, healthy animal as control; lanes 2–7, E2F3 protein appears to be overexpressed in all examined tumor samples.

**Figure 12:**
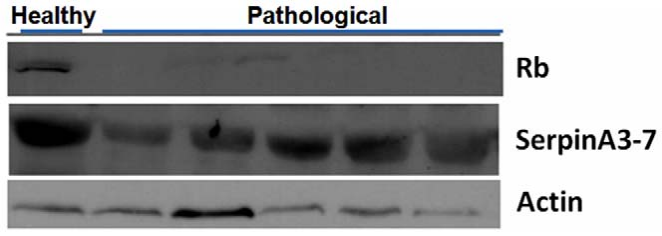
Expression of activated Capn3 in urothelial tumors. Western blot analysis to characterize the expression of pRb and constitutive SerpinA3-7 protein. A downregulation of pRb protein expression is severely evident in all tumor samples. The SerpinA3-7 protein was normally expressed in tumor samples.

To establish the expression levels of E2F3 protein, we performed a western blot analysis (Figure 11) and we observed that E2F3 was markedly overexpressed in tumor samples. Since E2F3 interacts with the retinoblastoma tumor suppressor protein (pRb), we also investigated pRb expression and detected a severe downregulation, to actual inactivation of pRb protein in the same cancer samples (Figure 12). Finally, we performed an investigation to validate the effective presence of SerpinA3-7 protein identified by a single peptide by means of proteomic analysis. We showed that the molecular complex obtained using an anti-E2F3 antibody was also composed of SerpinA3-7 (Figure 12).

## Discussion

Although various calpain substrate proteins are associated with carcinogenesis [2], the molecular identities of these substrates are largely unknown [26]. It has been suggested that Capn3 and the conventional calpains have common substrate specificities, as several proteins known to be potential targets of Capn3 appear the same substrates of conventional calpains [9], [26].

Recently, calpains have been shown to be involved in proteolysis of NORE1A, a potential Ras effector, and of Ras-association domain family 1 (RASSF1A) proteins. Downregulation of NORE1A and RASSF1A proteins might be involved in some carcinogenesis mechanisms [27]. Calpain 4 (Capn4) has been found to be associated with metastasis and recurrence of hepatocellular carcinoma (HCC). Therefore, it has been proposed that Capn4 might be a target for cancer therapy [28]. In addition, Calpain 6 (Capn6) expression was found increased in some uterine tumors [29].

High levels of Capn3 without any protease activity were detected in human melanoma cell lines. Therefore, it has been suggested that Capn3 may play a pro-apoptotic role in melanoma cells, and it could be a useful diagnostic marker for monitoring melanoma development and progression [30], [31].

In our cases, Capn3 expression was detected in twelve papillomavirus-positive neoplastic urothelial lesions. Capn3 protein was initially identified with nanoscale liquid chromatography coupled with tandem mass spectrometry (nano LC-MS/MS) in a co-immunoprecipitation experiment on E2F3. Capn3 expression was confirmed by reverse transcription polymerase chain reaction (RT-PCR) and its Ca^2+^-dependent proteolytic activity was assayed following ion exchange chromatography. Morphologically, Capn3 expression was documented by immunohistochemical examination. To our knowledge, this is the first study showing Capn3 activation in spontaneous oncogenesis in medical literature. We did not investigate whether any oncogenic proteins of BPV-2 could be involved in Capn3 activation. It has been shown that human papillomavirus (HPV) E7 oncoprotein is able to down-regulate pRb expression [32], since it binds to µ-calpain and activates its proteolytic activity, resulting in cleavage of pRb [6], [33]. Other mechanisms are likely to exist in animal cancers in which BPV-2 infection may play a central role. We have also shown that papillomavirus-associated urothelial cancers of cattle are characterized by an overexpression of sigma 2 receptors [34]. These receptors are known to play a crucial role in modulating cytoplasmic [Ca^2+^] in cancer cells [35]. It is reasonable to suggest that sigma 2 receptors may be involved in Capn3 activation, as it has been shown that even small increases of resting cytoplasmic [Ca^2+^] are responsible for Capn3 proteolytic activity [36], [37].

The role of the Capn3 protein in urothelial bladder cancer still remains to be elucidated. We have shown also E2F3 protein overexpression and a dramatic decrease of Rb protein in bladder cancers. Taken together our data allow us to suggest that pRb may be a calpain substrate also in bovine urothelial tumors. E2F3 levels are known to be regulated by pRb and have a crucial role in activating cell proliferation in human bladder cancers [21], [38]–[41]. As Capn 3 was only detected in molecular complexes immunoprecipitated with E2F3, it can be argued that the proteolitically active form of Capn3 protein might be directly involved in molecular pathways leading to the overexpression of E2F3 transcription factors, very likely via Rb protein degradation. Furthermore, Capn3 is known to be a regulator of the conventional calpain system [42]. Therefore, it cannot be excluded that Capn3 may play a role in regulating E2F3 protein expression in an indirect manner, as conventional µ-calpain is known to promote the degradation of Rb protein [6]. It has been suggested that inactivation of Rb pathway and overexpression of E2F3 are obligate events in some human bladder tumors [21]. Our suggestion appears to be strengthened by the proteomic profiles of the other complexes from the same samples, immunoprecipitated with antibodies, vs proteins known to play a role in BPV-2 infection, such as PDGF receptor beta [43]. No Capn3 was detected in them. Indeed, extracellular matrix components such as lumican and decorin were predominantly detected in these complexes (data not shown). Our observations appear to be consistent with the very interesting emerging evidence of a broader role of PDGFRs in tumor stromogenesis [44], and with the increasing interest in the role of decorin and lumican via the tyrosine kinase receptor family in cancer biology [45].

Further studies are needed to better understand the role of the calpain system in bladder carcinogenesis. If our results are validated by other studies, Capn3 will definitely appear to play an important role in the molecular pathway of bovine urinary bladder tumors. As a consequence, it may prove useful as a diagnostic biomarker for monitoring urothelial tumor development and progression and as a potential target for cancer therapy. Cattle suffering from urothelial tumors, whose incidence may be ∼90% in adult animals [20], [46], may serve as an animal model useful for gaining insight into new molecular pathways involved in naturally occurring bladder carcinogenesis and for evaluating *in vivo* potential new drugs against specific targets, or for proposing novel therapeutic strategies that are urgently needed nowadays [47]. It is also worth to remember that the establishment of reliable and reproducible animal models for bladder cancer remains an ongoing challenge, since developing therapeutic agents requires *in vivo* models [47]. Furthermore, the UICC Study Group suggested that biological models may still be trail blazing for the natural history of cancer, although molecular models have fostered an impressive progress over the last decades [48]. Finally, it is worthwhile noting that cattle has already been investigated and, it has been found to be a good animal model for several other human diseases [49], [50].
